# The first outbreak of feline panleukopenia virus infection in captive Pallas’s cats in Xining Wildlife Park

**DOI:** 10.3389/fvets.2024.1418553

**Published:** 2024-08-29

**Authors:** Jingjie Wei, Yuqi Shi, Xiaofei Wang, Shunfu He, Xinzhang Qi, Rong Lu, Yuhang Gao, ZiChen Liu, Yingyun Wang, Yuanheng Wu, Yipeng Jin

**Affiliations:** ^1^Department of Clinical Veterinary Medicine, College of Veterinary Medicine, China Agricultural University, Beijing, China; ^2^Qinghai Tibet Plateau Wildlife Park, Xining, Qinghai, China

**Keywords:** feline panleukopenia virus, Pallas’s cats, virus isolation and identification, pathogenicity, virus detection

## Abstract

**Introduction:**

In August 2021, an outbreak of Feline Panleukopenia Virus (FPV) was observed in four 3-month-old Pallas’ cats at Xining Wildlife Park. Despite timely intervention, the Pallas’cat cubs continued to experience clinical symptoms including diarrhea, seizures, and decreased white blood cell count, and all four cats died.

**Methods:**

FPV clinical suspicions were initially confirmed by positive Polymerase Chain Reaction (PCR) testing. Pathological and immunohistochemical examinations (IHC) were performed on some organs, and the results showed that, encephalitis, viral enteritis, and splenitis occurred.

**Results:**

The virus replicates extensively in the cytoplasm of lymphocytes and macrophages in the lamina propria of the small intestine mucosa. A strain of FPV was successfully isolated and culture in CRFK cells. Through molecular identification, sequence analysis, and phylogenetic analysis of the VP2 gene in this strain, we have revealed the presence of a novel synonymous mutation. From July to December 2021, surveillance on stray cats and susceptible wildlife at Xining Wildlife Park indicated widespread FPV transmission.

**Discussion:**

The findings highlight the urgent need for ongoing epidemiological monitoring and active disinfection measures to prevent FPV transmission in wildlife parks.

## Introduction

Feline panleukopenia virus (FPV) is a highly infectious non enveloped DNA virus belonging to the genus Protoparvovirus of the Parvoviridae family, characterized by a single stranded genome. It has strong resistance to environmental factors and can last for months to years (1–3). The incubation period of FPV virus is 2–9 days, and it can cause feline leukopenia, a disease characterized by severe reduction in circulating white blood cell count and enteritis. Clinical manifestations include fever, anorexia, vomiting, diarrhea, and drowsiness (4). FPV poses a major threat to young animals because of its high incidence rate and mortality in this population. FPV can not only infect domestic cats, but more and more wild animals have been detected as hosts of parvovirus, including raccoons, foxes, and minks (5). Previous research reports have reported the detection of FPV sequences in the tissues of African wildcats, as well as in the feces of cheetahs and honey badgers (6). The FPV whole-genome is approximately 5Kb long and consists of a middle coding region and unique palindromic structures at both ends. The palindromic sequence base pairs to form a hairpin double stranded structure, and the coding regions encode non structural proteins NS (NS1 and NS2) and viral structural proteins VP (VP1 and VP2), respectively. VP2 protein is the main antigenic protein encoded by FPV, which plays an important role in immune response, recognition of receptors, and infection of tissues. The sequencing and phylogenetic analysis of the VP2 gene and the full-length genome of FPV will provide information on the main antigen types of FPV.

The Pallas’s cat, a second-level protected wild animal in China, faces challenges in artificial breeding due to its rare numbers. The main habitat of the Pallas’s cat is in central Asia. It is the only purebred captive member of the Felidae family, belonging to the high-altitude subspecies, located in the Xining Wildlife Park in Qinghai Province, China. Until now, there have been no reports of Pallas’s cat being infected with FPV.

**Figure 1:**
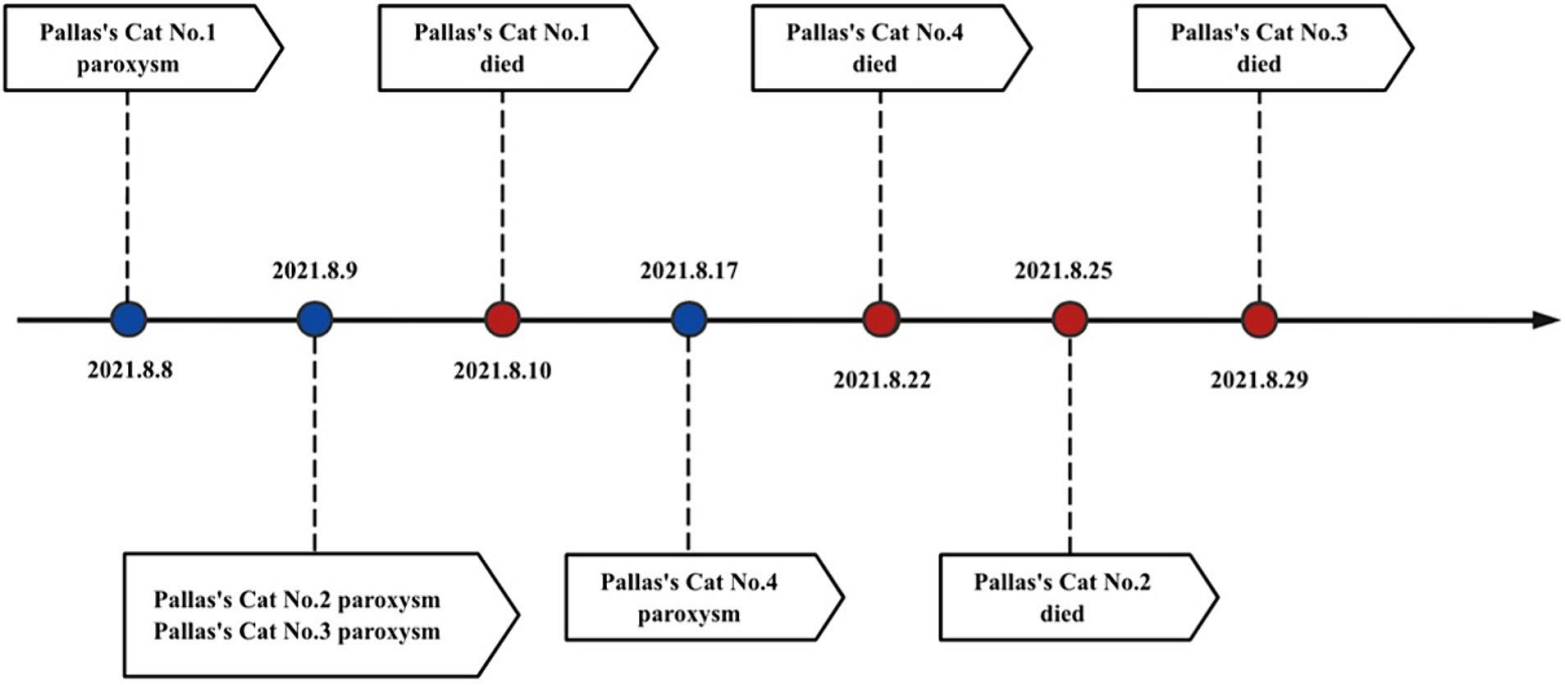
Time axis of FPV of Pallas’s cat.

In 2021, four Pallas’s cat cubs in the park experienced symptoms such as vomiting, diarrhea, and drowsiness, with white blood cell counts less than 5.5–15.4 × 10^9^/L Among them, two quickly developed symptoms of coma, followed by seizures, and subsequently died (see Supplementary Table S1 for details and Figure 1 for the disease progression timeline) (7). Feces and throat swabs collected during the early onset of the disease were subjected to nucleic acid amplification sequencing of the FPV VP2 gene fragment, and the 841 nt fragment closely homologous to FPV-SH2003 was examined (similarity 99.51%, specific sequence see Appendix 1).

Despite timely intervention with medication and feeding of prescribed canned food, all four cubs still did not survive. This marks the world’s first reported case of Pallas’s cat cubs infected with FPV. We conducted studies on the virological characteristics and pathogenicity of the virus, and monitored the prevalence in the park.

## Methods

### Sample collection

During the treatment of affected Pallas’s cats, we systematically collected feces and throat swabs. Post-mortem examinations were performed on two young male Pallas’s cats, with a comprehensive analysis of major organs including the brain, spleen, and small intestine.

### Virus isolation and identification

To assess virus virulence, feces of four Pallas’cat cubs were used to isolate and identify the viral strain for assessing. The entire process involved mixing feces with PBS solution, centrifuging at 7,000 rpm for 20 min. Then, 0.5 mL of the supernatant was added to an equal volume of chloroform, shaken, and centrifuged at 4,000 rpm for 25 min. The supernatant was transferred to a new centrifuge tube and centrifuged again at 12,000 rpm for 10 min to obtain the virus fluid. For virus isolation on cell culture, the virus fluid was inoculated into CRFK culture plates, gently mixed, and incubated in cell culture medium at 37°C with 5% CO_2_ concentration. Cell cytopathic changes were observed after blind passage for 3–4 generations. Nucleic acid extraction was performed for whole-genome amplification, using specific primers outlined in Supplementary Table S2, ddH2O served as the negative control. The amplification system (total volume of 40 μL) is detailed in Supplementary Table S3, and reaction conditions are provided in Supplementary Table S4. PCR products were extracted for agarose gel electrophoresis and sequencing, the presence of a specific amplified fragment of approximately 511 bp, as confirmed by agarose gel electrophoresis, was considered a positive result.

### Hemagglutination test

Mix fresh pig blood with Asperger’s solution and prepare a 1% volume fraction red blood cell suspension using PBS. The microplate method was used for the hemagglutination test on a 96-well V-shaped plate. Virus fluid was serially diluted twofold from well 1 to well 11, with 25 μL drawn and discarded. Well 12 was used as the negative control. Add 25 μL to each hole μ Mix 1% pig red blood cell suspension well, place at 4°C for 1–2 h, room temperature for 3 min. A 50% red blood cell agglutination was considered as the hemagglutination titer for the viral strain.

### Amplification and sequencing of VP2 gene

Utilizing the preliminary sequencing results of nucleic acid analysis, the conserved region of the VP2 segment with the highest nucleotide homology in the FPV strain was identified through BLAST in the NCBI database (GenBank: MW8111871). Specific amplification primers are detailed in Supplementary Table S5, amplification systems are shown in Supplementary Table S3 (total volume 40 μL), reaction conditions are shown in Supplementary Table S4, and sequencing primers are shown in Supplementary Table S6.

### Hematoxylin and eosin and immunohistochemistry

We performed necropsies on two of the deceased Pallas’s cat cubs (both males). Major tissues and organs including spleen, brain, intestines were collected, fixed with 4% paraformaldehyde, embedded in paraffin, and sliced into 5 μm sections. Hematoxylin and eosin (H.E.) staining were performed for microscopic examination. Immunohistochemical analysis utilized mouse monoclonal antibodies against canine parvovirus (ab59832, Abcam, Cambridge, UK) and horseradish peroxidase (HRP) in the jejunum. Tissue slices were observed under a light microscope, brownish-yellow positive signals observed in the cytoplasm of lymphocytes and macrophages in tissue sections were considered indicative of FPV presence.

### Virus monitoring

We collected feces and throat swabs and blood samples from 19 stray cats inside and around the park. Park staff were tasked with regularly using sterile swabs to collect environmental samples from key areas, such as the soles of shoes, sealed animal products, and veterinary hospital treatment equipment. Animal samples including feces and urine from animal cages, as well as saliva from easily manageable animals, were also collected. Mix the sample with PBS solution and centrifuge at 7,000 rpm for 20 min. Then, add 0.5 mL of the supernatant to an equal amount of chloroform, shake well, and centrifuge at 4,000 rpm for 25 min. Transfer the supernatant to a new centrifuge tube and centrifuge again at 12,000 rpm for 10 min to obtain the virus solution. For blood samples, centrifugation was performed at 5,000 rpm for 3 min, and then 10 μL of the supernatant was diluted according to the instructions of the FPV immunofluorescence antibody test paper. Subsequently, 80 μL of buffer solution was dropped onto the test paper, and the paper was inserted into a time-resolved fluorescence quantitative detection instrument, left to stand for 10 min, and then checked for results. Swab samples were thoroughly shaken in 200 μL of sterile saline for washing. Then, 1.5 μL was added into the FPV nucleic acid rapid detection tube, and nucleic acid detection was performed using a qPCR instrument. Specific amplification primers are detailed in Supplementary Table S3, and reaction conditions are provided in Supplementary Table S7.

## Results

### Hemagglutination test and cellular response to FPV infection

**Figure 2:**
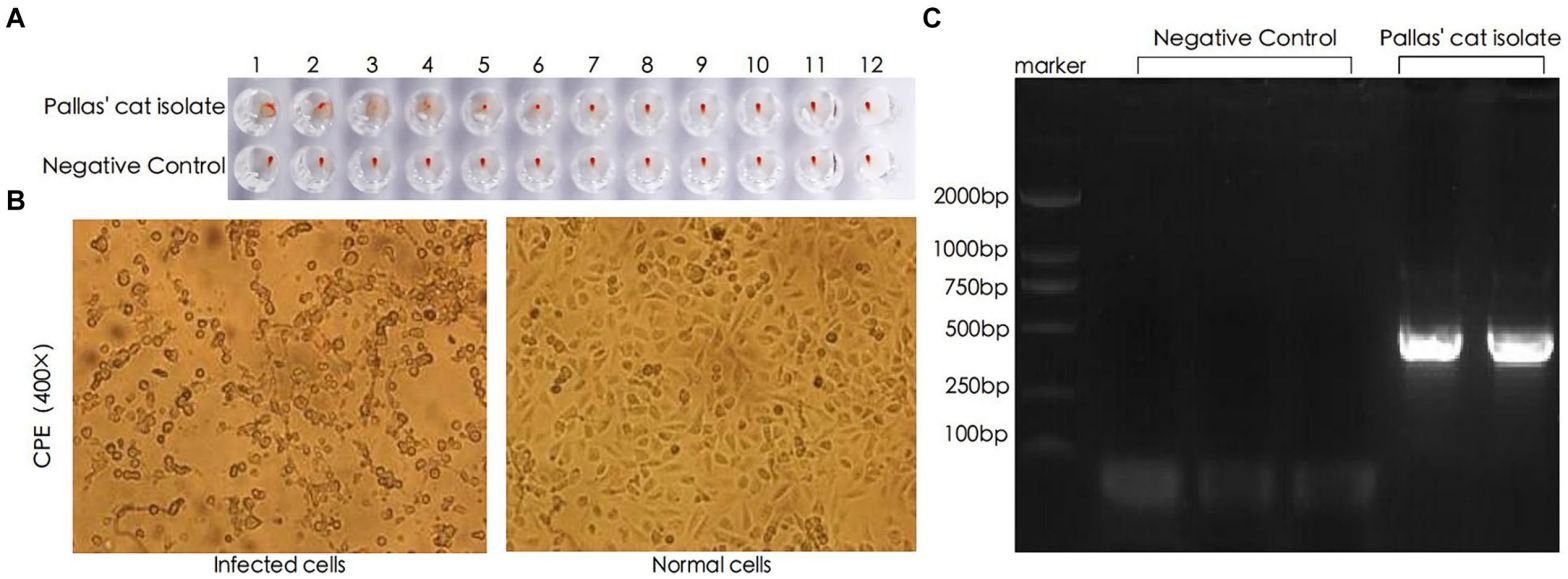
Identification of FPV through HA, CPE, and PCR. **(A)** Hemagglutination test results. **(B)** Pathological changes observed in CRFK (Cat Renal Fibroblast Kidney) cells 20 h after inoculation with the viral strain (400×). **(C)** PCR analysis of viral DNA genome.

The hemagglutination test results showed that the clotting titer of this FPV strain was 2^7^, as depicted in Figure 2A. Following virus infection of CRFK cells, significant cytopathic effect was observed 20 h post-inoculation. The cells exhibited typical cytopathic changes such as cell rounding, reduced intercellular contacts, and elongation, characteristic of small virus lesions, whereas the control group cells maintained normal morphology (5, 8), as shown in Figure 2B. The nucleic acid amplification results, confirmed by agarose gel electrophoresis, showed an amplified fragment size of approximately 511 bp, consistent with the expected results, as illustrated in Figure 2C. The recovered amplification products were sequenced using the same primers, and the results matched the FPV genome.

### Genomic sequencing and phylogenetic analysis of the FPV strain

**Figure 3:**
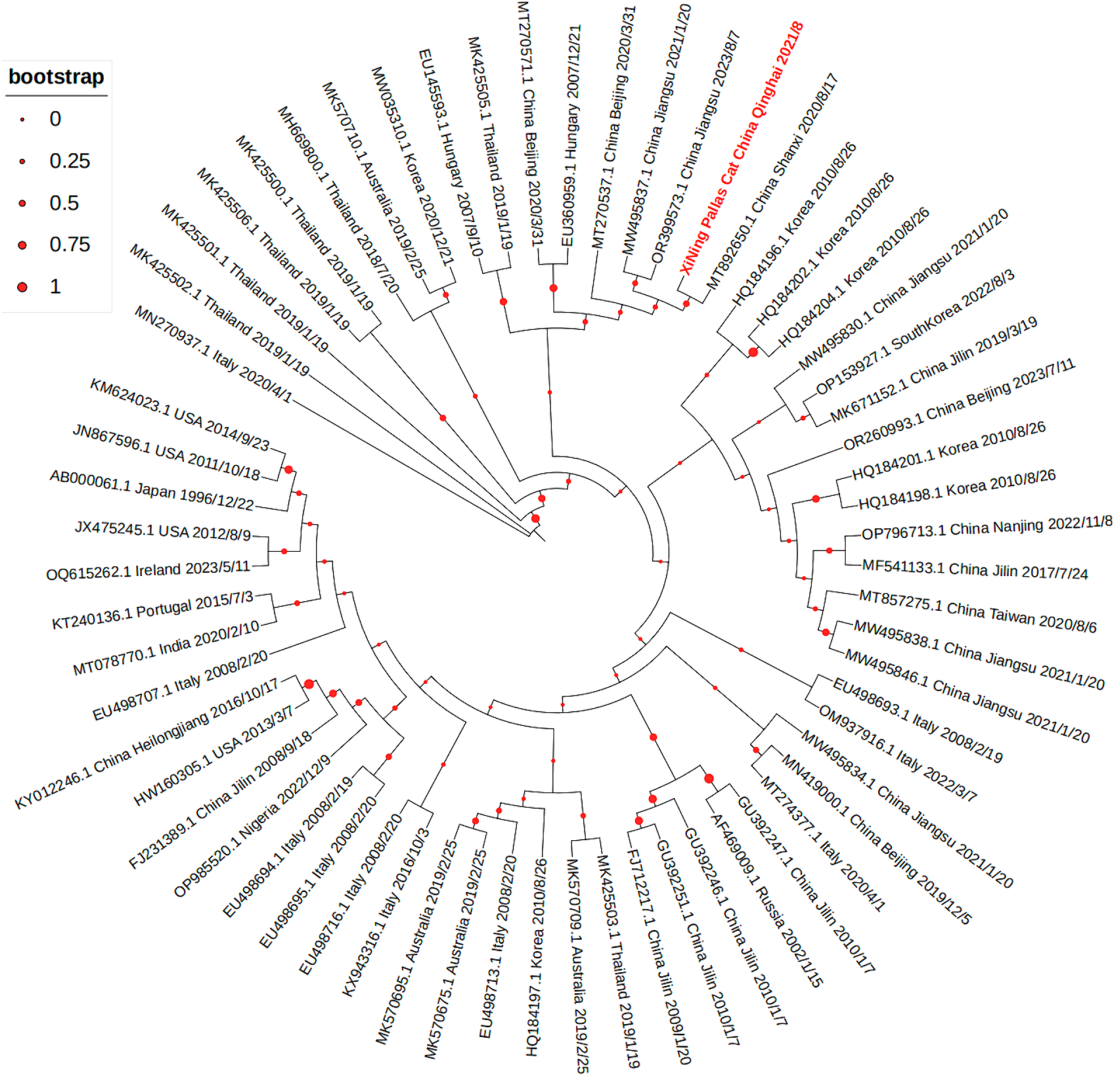
The phylogenetic tree of VP2 gene shows that the VP2 sequence of the Pallas’s cat isolate is closely related to multiple prevalent strains in China.

The sequencing results revealed that the virus’s entire genome length was 4,679 nt, with the specific sequence detailed in Appendix 2. The VP2 fragment, spanning 1,755 nt, encodes 584 amino acids, and its specific nucleic acid sequence is provided in Appendix 3, while the amino acid sequence is presented in Appendix 4. Using the NCBI database, the VP2 sequence was translated and its amino acid sequence was compared with the FPV standard strain. The sequencing analysis of the FPV VP2 sequence of the Pallas’cat showed 99.94% nucleotide homology with the domestic popular strain, and it was consistent with the FPV standard strain CU-4 at 5 key amino acid sites (80 K, 93 K, 103 V, 323D, and 568A), with a homology of 99.49% Upon comparing with other uploaded FPV sequences, a notable change was observed at the 756th base, which had not been seen in other FPV strains. The change from A to G, however, resulted in no alteration in amino acid types, classifying it as a synonymous mutation. Using MEGA 7.0 software, a phylogenetic tree was constructed through the neighbor-joining method to identify common VP2 sequences and VP2 sequences of Pallas’s cat isolates, as illustrated in Figure 3. The representation “XiNing Pallas’cat China Qinghai 2021/8” in the figure signifies the VP2 sequence of the Pallas’s cat isolate.

### Pathological findings and immunohistochemical analysis in Pallas’s cat tissues

**Figure 4:**
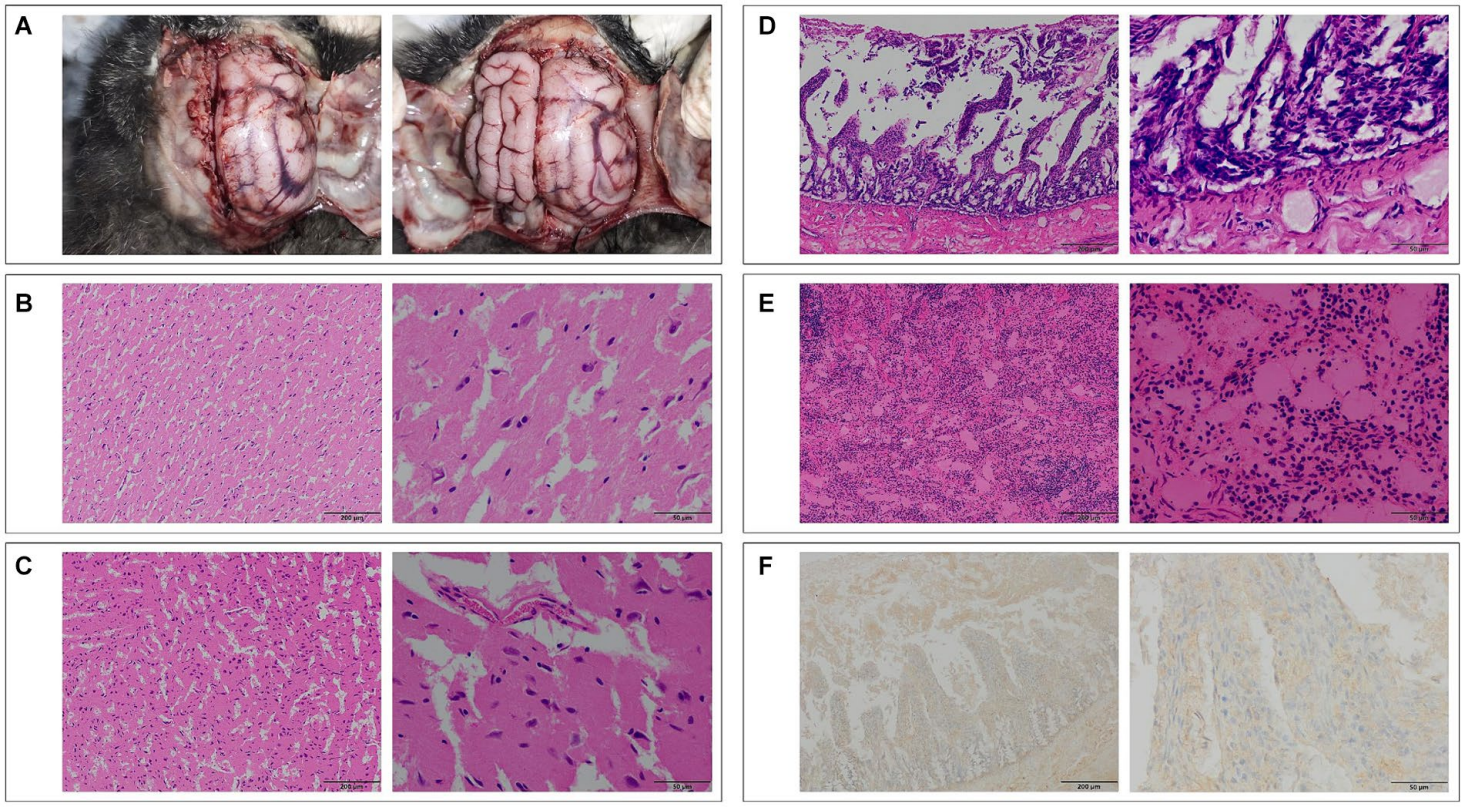
**(A)** Top view of the brain of Pallas’s cat No. 1, showing significant edema (Top view, head facing upward). **(B)** Pathological section of the right hemisphere of the brain of Pallas’s cat No. 1, showing mild neurophagocytosis (100×, left) and edema (400×, right). **(C)** Pathological section of the cerebellum of Pallas’s cat No. 1, showing edema (100×, left; 400×, right). **(D)** Pathological examination of the jejunum of Pallas’s cat No. 4, showing vascular congestion, edema, villi rupture, and lymphocyte infiltration (100×, left; 400×, right). **(E)** Pathological examination of the spleen of Pallas’s cat No. 4, showing lymphocyte depletion, capillary dilation and congestion, and red pulp area expansion (100×, left; 400×, right). **(F)** Immunohistochemical examination of the jejunum of Pallas’s cat No. 4, showing positive FPV antigen signals in the cytoplasm of lymphocytes and macrophages (100×, left; 400×, right).

During the initial dissection of the Pallas’s cat brain, signs of edema were evident, with some gyriand sulci disappearing. Pathological sections revealed mild neurophagocytosis in the right brain, accompanied by edema in the cerebellum and brainstem (5, 8). Pathological examination of the jejunum revealed submucosal congestion and edema, villus rupture, and lymphocyte infiltration in the lamina propria. Spleen examination showed lymphocyte depletion, capillary dilation and congestion, expansion of the red pulp, and contraction of the white pulp. Lmmunohistochemical analysis of Pallas’s cat detected the presence of viral antigen, indicated by the brownish yellow positive signal primarily in the cytoplasm of lymphocytes and macrophages in the laminapropria of the jejunum mucosa, as depicted in Figure 4 (9, 10).

### Epidemiological survey of FPV in Xining Wildlife Park: population and environmental assessment

**Figure 5:**
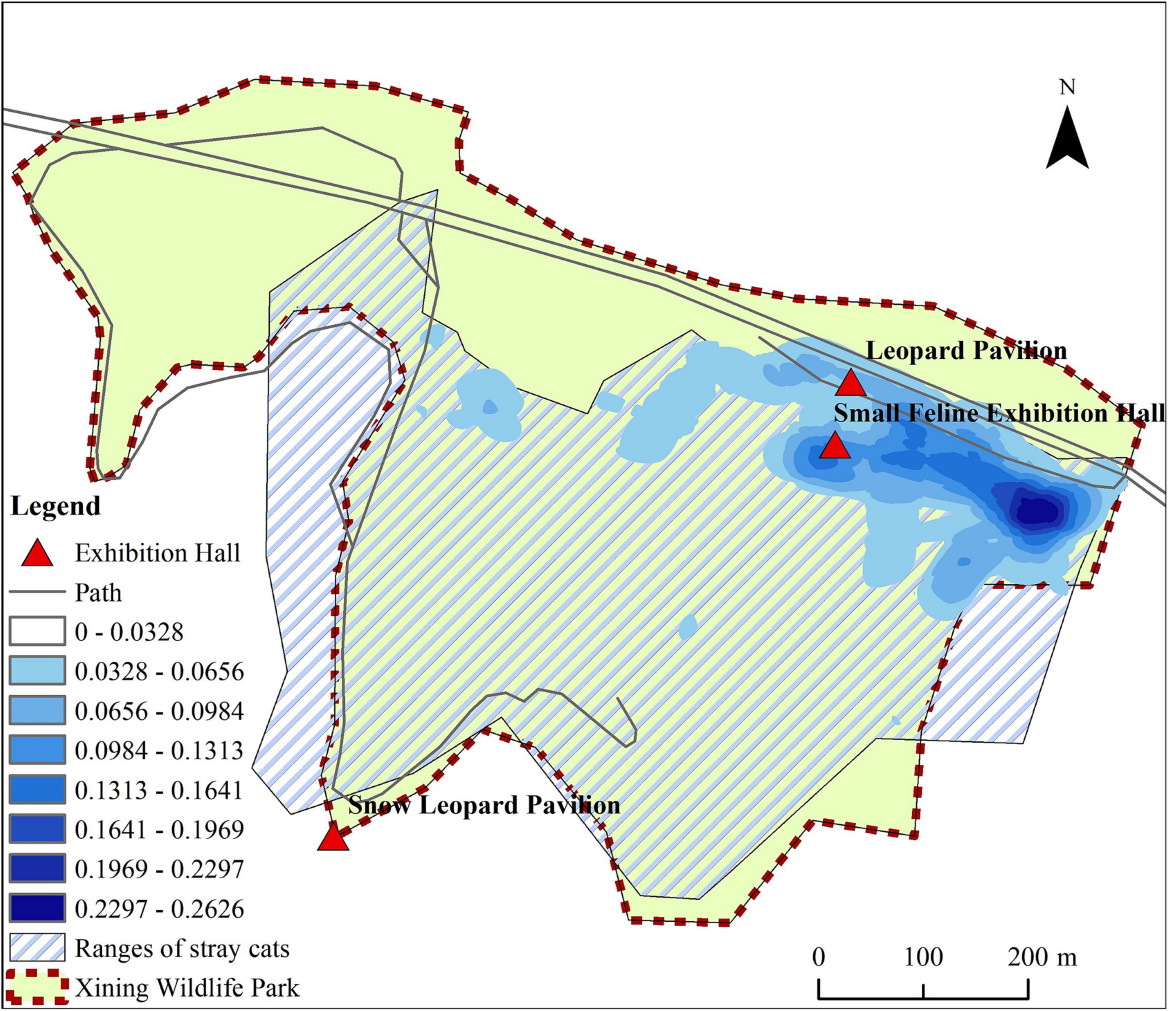
The main activity areas and frequency of stray cats in Xining Wildlife Park.

In the epidemiological investigation of the FPV epidemic among Pallas’s cats in Xining Wildlife Park, FPV detection results were obtained from both the population and the environment of stray cats. The main activity areas and frequency of stray cats in Xining Wildlife Park were shown in Figure 5. Between July and September 2021, a total of 11 stray cats were captured within Xining Wildlife Park, revealing a seropositive rate of 72.72% and a viral DNA detection rate of 54.55%. An additional 8 stray cats captured around the park exhibited a 100% seropositive rate and a 47.37% viral DNA detection rate. Environmental samples were collected and tested from various locations within the park, including the Parrot Pavilion, Leopard Pavilion, Small Feline Exhibition Hall, Snow Leopard Pavilion, Monsters Pavilion, Lesser Panda Pavilion, Veterinary Hospital, and ingredient rooms. The overall positive rate for the virus in these environmental samples was 40.67%. Notably, the Leopard Pavilion had 81 positive samples, constituting 10.01% of the total samples and 24.62% of the total positive samples. Additionally, the environment of adult Pallas’s cats and Pallas’s cat cubs had 84 positive samples, accounting for 10.38% of the total sample count and 25.53% of the positive sample count. The combined positive samples from the Leopard Pavilion and Pallas’s cat species represented 50.15% of the total positive samples, indicating the widespread presence of FPV in these specific environments. Throughout the monitoring period, positive samples were detected in all venues. Among animal samples, positive results were found in 5 snow leopard feces, 6 snow leopard urine samples, 2 lion feces, 1 bear feces, and 1 leopard cat feces. The overall positive rate for fecal samples was 7%, and for urine samples, it was 13.6%. The evidence of such a high positivity rate in environmental samples is unusual, and these findings emphasize the universality of FPV in the population and environmental samples of Xining Wildlife Park.

## Discussion

This study represented the first documented instance of FPV infection in Pallas’s cat cubs. We investigated the pathogenicity and origin of FPV strains using methods such as virus isolation on cell culture, hemagglutination assay, PCR, hematoxylin–eosin staining, and immunohistochemistry.

The initial clinical signs observed in the affected Pallas’s cats were consistent with typical FPV signs (11). FPV diagnosis is typically based on clinical symptoms, leukopenia in complete blood cell counts, and detection of fecal viral antigen or PCR testing. The pathological findings we observed in the three tissues of Pallas’s cats were consistent with those observed in domestic and wild cats, highlighting the widespread impact of FPV.

FPV primarily replicates in mitotically active tissues, causing leukopenia by invading early stem cells and lymphocytes in the lymph and spleen of feline animals. It damages intestinal mucosal crypt cells, leading to intestinal villi damage, poor absorption, increased permeability, and diarrhea (9, 12, 13). Therefore, selecting spleen and intestinal tissues for pathological examination reveals more evident pathological changes. Cats infected with FPV may exhibit central nervous system signs and neurodegenerative morphological changes (14, 15). To study FPV-induced pathological changes in Pallas’s cat tissues, we dissected cadavers, collecting major organs for H.E staining and immunohistochemistry (16). The pathological changes mirrored those in various wild feline organisms and domestic cats (11, 17–19). H.E staining results confirmed FPV’s impact, and immunohistochemistry revealed abundant FPV in the cytoplasm of lymphocytes and macrophages in the small intestine, indicating FPV proliferation and invasion, ultimately leading to diarrhea.

We measured the virus titer using a hemagglutination test, and the results were consistent with the HA test of the domestic epidemic strain (20). There is clear evidence that FPV can infect feline animals such as snow leopards (6, 21). Comparing the amino acid sequences of isolated strains with commercial vaccine strains, differences in amino acid residues at positions 91, 232, and 562 may alter the recognition of neutralizing antibodies, leading to immune evasion (22). Compared with the commercial vaccine strain, the nucleotide and amino acid homology was 99.37 and 99.49%, respectively. Amino acid residue differences at positions 91, 232, and 562 may alter neutralizing antibody recognition, potentially leading to immune evasion. The change at V232I resembles CPV-2, suggesting an evolutionary link between FPV and CPV-2, possibly impacting vaccine efficacy. The amino acid sequence matches the 2019 South China tiger fecal isolate, implying differing evolutionary trends between wild and domestic feline populations, necessitating more wild feline FPV sequences for conclusive evidence (23, 24).

**Figure 6:**
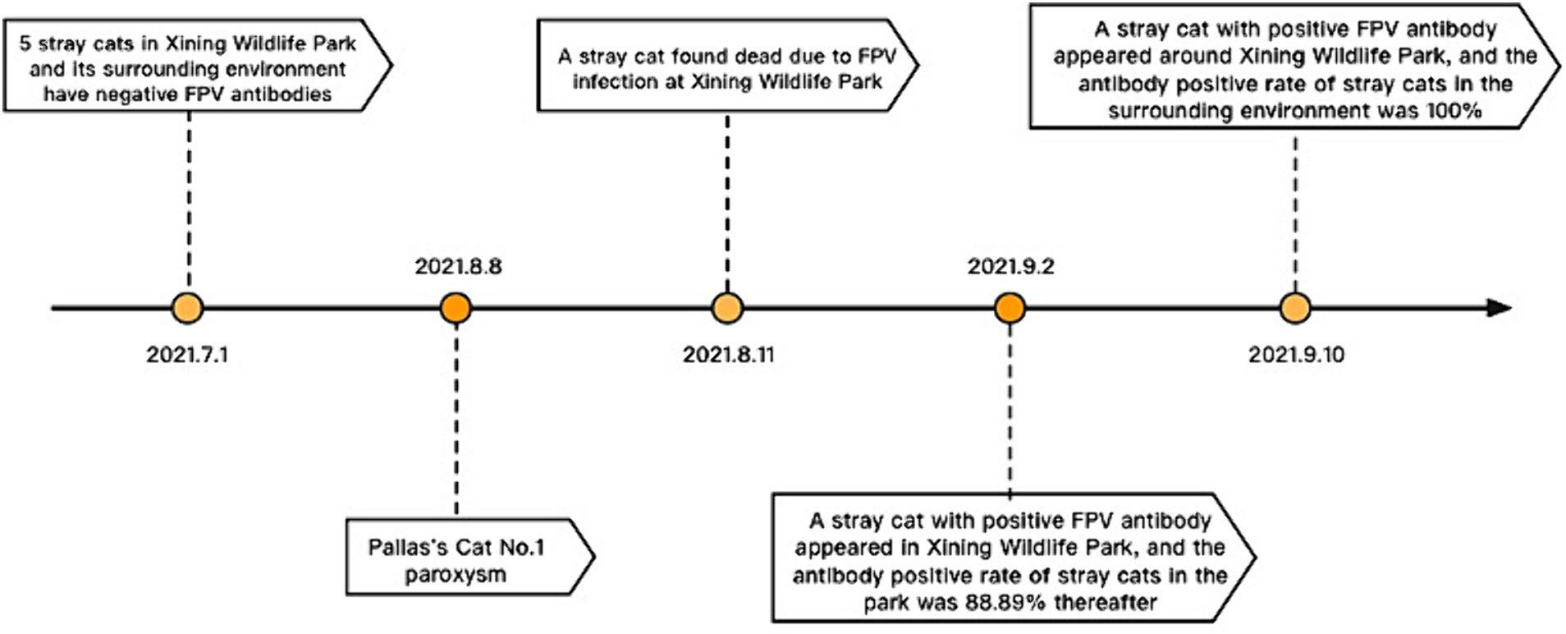
Time axis of epidemiological.

After the Pallas’s cat FPV outbreak, essential steps in virus tracing, epidemic distribution, monitoring, and warning are crucial. The European Association of Zoological and Wildlife Veterinary Societies emphasized preventive measures, including trapping and sterilization to hinder stray cats’ contact with wild felines in the park (25, 26). Multiple studies also highlight that viral infectious diseases in zoo wild cats often originate from stray cats (11, 25–27). Epidemiological monitoring in July 2021 found no FPV in captured stray cats before the outbreak on August 8. However, three days after the first Pallas’cat fell ill, a stray cat was discovered and tested positive for the anal swab using FPV antigen rapid diagnostic test strips. (Figure 6). Subsequent tests revealed a high number of cats with anti-FPV antibodies in the park (88.89%) and in the surrounding residential areas (100%), indicating the likely spread of FPV from these areas to the park. Stray cats are probable spreaders in this epidemic, highlighting the need for comprehensive preventive measures. Given the current situation, we will continue to monitor the FPV nucleic acid in environmental samples, which provides valuable reference for planning virus disinfection and disinfection work in the park.

## Conclusion

This study highlighted the FPV infection in juvenile captive Pallas’s Cats. Splenic lymphocyte failure and neurophagocytic cell phenomenon was found. Virus was detected in lymphocytes and macrophages of the jejunal mucosa. A new synonymous mutation was found in the VP2 gene of an FPV isolate, which is closely related to multiple prevalent strains in China. From July to December 2021, FPV spread widely among stray cats and environment of captive wildlife. This emphasizes the need for ongoing epidemiological monitoring in Xining Wildlife Park to avert larger-scale FPV outbreaks and wild feline population decline.

## Data Availability

The original contributions presented in the study are publicly available. This data can be found here: https://doi.org/10.6084/m9.figshare.26779525.
