# Supplementary material for: The first outbreak of feline panleukopenia virus infection in captive Pallas’s cats in Xining Wildlife Park
Source: Front Vet Sci. 2024 Aug 29;11:1418553. doi: 10.3389/fvets.2024.1418553 (PMC11391486; doi:10.3389/fvets.2024.1418553)
Supplement: Supplementary file 1 [file Data_Sheet_1.docx]

Appendix

Appendix 1: Preliminary identification results of VP2 sequence of Pallas's cats pharyngeal swab FPV CAAGCAGCAGATGGTGATCCAAGATATGCATTTGGTAGACAACATGGTCAAAAAACTACTACAACAGGAGAAACACCTGAGAGATTTACATATATAGCACATCAAGATACAGGAAGATATCCAGAAGGAGATTGGATTCAAAATATTAACTTTAACCTTCCTGTAACAAATGATAATGTATTGCTACCAACAGATCCAATTGGAGGTAAAACAGGAATTAACTATACTAATATATTTAATACTTATGGTCCTTTAACTGCATTAAATAATGTACCACCAGTTTATCCAAATGGTCAAATTTGGGATAAAGAATTTGATACTGACTTAAAACCAAGACTTCATGTAAATGCACCATTTGTTTGTCAAAATAATTGTCCTGGTCAATTATTTGTAAAAGTTGCGCCTAATTTAACAAATGAATATGATCCTGATGCATCTGCTAATATGTCAAGAATTGTAACTTACTCAGATTTTTGGTGGAAAGGTAAATTAGTATTTAAAGCTAAACTAAGAGCATCTCATACTTGGAATCCAATTCAACAAATGAGTATTAATGTAGATAACCAATTTAACTATGTACCAAATAATATTGGAGCTATGAAAATTGTATATGAAAAATCTCAACTAGCACCTAGAAAATTATATTAACATACTTACTATGTTTTTATGTTTATTACATATTAACTAGCACCTAGAAAATTATATTAATATACTTACTATGTTTTTATGTTTATTACATATTATTTTAAGATTAATTAAATTACAGCATAGAAATATTGTACTTGTATTTGATATAGGATTTAAAGGTGTTTTTTATGGGGTTAAACCTTCACAACATGGG

Appendix 2: Whole genome of FPV Pallas's cats isolate

GTGGCGGGCTAATTGTGGGCGTGGTTAAAGGTATAAAAGACAAACCATAGACCGTTACTGACATTCGCTTCTTGTCTTTGACAGAGTGAACCTCTCTTACTTTGACTAACCATGTCTGGCAACCAGTATACTGAGGAAGTTATGGAGGGAGTAAATTGGTTAAAGAAACATGCAGAAGATGAAGCATTTTCGTTTGTTTTTAAATGTGACAACGTCCAACTAAATGGAAAGGATGTTCGCTGGAACAACTATACCAAACCAATTCAAAATGAAGAGCTAACATCTTTAATTAGAGGAGCACAAACAGCAATGGATCAAACCGAAGAAGAAGAAATGGACTGGGAATCGGAAGTTGATAGTCTCGCCAAAAAGCAAGTACAAACTTTTGATGCATTAATTAAAAAATGTCTTTTTGAAGTCTTTGTTTCTAAAAATATAGAACCAAATGAATGTGTTTGGTTTATTCAACATGAATGGGGAAAAGATCAAGGCTGGCATTGTCATGTTTTACTTCATAGTAAGAACTTACAACAAGCAACTGGTAAATGGCTACGCAGACAAATGAATATGTATTGGAGTAGATGGTTGGTGACTCTTTGCTCGGTAAACTTAACACCAACTGAAAAGATTAAGCTCAGAGAAATTGCAGAAGATAGTGAATGGGTGACTATATTAACATACAGACATAAGCAAACAAAAAAAGACTATGTTAAAATGGTTCATTTTGGAAATATGATAGCATATTACTTTTTAACAAAGAAAAAAATTGTCCACATGACAAAAGAAAGTGGCTATTTTTTAAGTACTGATTCTGGTTGGAAATTTAACTTTATGAAGTATCAAGACAGACATACTGTCAGCACACTTTACACTGAACAAATGAAACCAGAAACCGTTGAAACCACAGTGACGACAGCACAGGAAACAAAGCGCGGGAGAATTCAAACTAAAAAGGAAGTGTCAATCAAATGTACTTTGCGGGACTTGGTTAGTAAAAGAGTAACATCACCTGAAGACTGGATGATGTTACAACCAGATAGTTATATTGAAATGATGGCACAACCAGGAGGTGAAAATCTTTTAAAAAATACACTTGAAATTTGTACTTTGACTTTAGCAAGAACAAAAACAGCATTTGAATTAATACTTGAAAAAGCAGATAATACTAAACTAACTAACTTTGATCTTGCAAATTCTAGAACATGTCAAATTTTTAGAATGCACGGATGGAATTGGATTAAAGTTTGTCACGCTATAGCATGTGTTTTAAATAGACAAGGTGGTAAAAGAAATACAGTTCTTTTTCATGGACCAGCAAGTACAGGAAAATCTATTATTGCTCAAGCCATAGCACAAGCTGTGGGTAATGTTGGTTGCTATAATGCAGCGAATGTAAATTTTCCATTTAATGACTGTACCAATAAAAATTTAATTTGGGTTGAAGAAGCTGGTAACTTTGGTCAACAAGTTAATCAATTTAAAGCAATTTGTTCTGGACAAACAATTAGAATTGATCAAAAAGGTAAAGGAAGTAAGCAAATTGAACCAACTCCAGTAATTATGACAACTAATGAAAATATAACAATTGTAAGAATTGGATGTGAAGAAAGACCTGAACATACACAACCAATAAGAGACAGAATGTTGAACATTAAGTTAGTATGTAAGCTTCCAGGAGACTTTGGTTTGGTTGATAAAGAAGAATGGCCTTTAATATGTGCATGGTTAGTTAAACATGGTTATGAATCAACCATGGCTAACTATACACATCATTGGGGAAAAGTACCAGA ATGGGATGAAAACTGGGCGGAGCCTAAAATACAAGAAGGTATAAATTCACCAGGTTGCAAAGACTTAGAGACACAAGCGGCAAGCAATCCTCAGAGTCAAGACCAACTTCTAACTCCTCTGACTCCGGACGTAGTGGACCTTGCACTGGAACCGTGGAGTACTCCAGATACGCCTATTGCAGAAACTGCAAATCAACAATCAAACCAACTTGGCGTTACTCACAAAGACGTGCAAGCGAGTCCGACGTGGTCCGAAATAGAGGCAGACCTGAGAGCCATCTTTACTTCTGAACAATTAGAAGAAGATTTTCGAGACGACTTGGATTAAGGTACGATGGCACCTCCGGCAAAGAGAGCCAGGAGAGGTAAGGGTGTGTTAGTAAAGTGGGGGGAGGGGAAAGATATAATAACTTAACTAAGTATGTGTTTTTTTACAGGACTTGTGCCTCCAGGTTATAAATATCTTGGGCCTGGGAACAGTCTTGACCAAGGAGAACCAACTAACCCTTCTGACGCCGCTGCAAAAGAACACGACGAAGCTTACGCTGCTTATCTTCGCTCTGGTAAAAACCCATACTTATACTTTTCGCCAGCAGATCAACGCTTTATAGATCAAACTAAGGACGCTAAAGATTGGGGGGGGAAAATAGGACATTATTTTTTTAGAGCTAAAAAAGCAATTGCTCCAGTATTAACTGATACACCAGATCATCCATCAACATCAAGACCAACAAAACCAACTAAAAGAAGTAAACCACCACCTCATATTTTCATCAATCTTGCAAAAAAAAAAAAAGCCGGTGCAGGACAAGTAAAAAGAGACAATCTTGCACCAATGAGTGATGGAGCAGTTCAACCAGACGGTGGTCAACCTGCTGTCAGAAATGAAAGAGCTACAGGATCTGGGAACGGGTCTGGAGGCGGGGGTGGTGGTGGTCTGGGGGGTGTGGGGATTTCTACGGGTACTTTCAATAATCAGACGGAATTTAAATTTTTGGAAAACGGGTGGGTGGAAATCACAGCAAACTCAAGCAGACTTGTACATTTAAATATGCCAGAAAGTGAAAATTATAAAAGAGTAGTTGTAAATAATATGGATAAAACTTCAGTTAAAGGAAACATGGCTTTAGATGATACTCATGTACAAATTGTAACACCTTGGTCATTGGTTGATGCAAATGCTTGGGGAGTTTGGTTTAATCCAGGAGATTGGCAACTAATTGTTAATACTATGAGTGAGTTGCATTTAGTTAGTTTTGAACAAGAAATTTTTAATGTTGTTTTAAAGACTGTTTCAGAATCTGCTACTCAGCCACCAACTAAAGTTTATAATAATGATTTAACTGCATCATTGATGGTTGCATTAGATAGTAATAATACTATGCCATTTACTCCAGCAGCTATGAGATCTGAGACATTGGGTTTTTATCCATGGAAACCAACCATACCAACTCCATGGAGATATTATTTTCAATGGGATAGAACATTAATACCATCCCATACTGGAACTAGTGGCACACCAACAAATGTATATCATGGTACAGATCCAGATGATGTTCAATTTTATACTATTGAAAATTCTGTACCAGTGCACTTACTAAGAACAGGTGATGAATTTGCTACAGGAACATTTTTTTTTGATTGTAAACCATGTAGACTAACACATACATGGCAAACAAATAGAGCATTGGGCTTACCACCATTTTTAAATTCTTTGCCTCAATCTGAAGGAGCTACTAACTTTGGTGATATAGGAGTTCAACAAGATAAAAGACGTGGTGTAACTCAAATGGGAAATACAGACTATATTACTGAAGCTACTATTATGAGACCAGCTGAGGTTGGTTATAGTGCACCATATTATTCTTTTGAAGCATCTACACAAGGGCCATTTAAAACACCTATTGCAGCAGGACGGGGGGGAGCGCAAACAGATGAAAATCAAGCAGCAGATGGTGATCCAAGATATGCATTTGGTAGACAACATGGTCAAAAAACTACTACAACAGGAGAAACACCTGAGAGATTTACATATATAGCACATCAAGATACAGGAAGATATCCAGAAGGAGATTGGATTCAAAATATTAACTTTAACCTTCCTGTAACAAATGATAATGTATTGCTACCAACAGATCCAATTGGAGGTAAAACAGGAATTAACTATACTAATATATTTAATACTTATGGTCCTTTAACTGCATTAAATAATGTACCACCAGTTTATCCAAATGGTCAAATTTGGGATAAAGAATTTGATACTGACTTAAAACCAAGACTTCATGTAAATGCACCATTTGTTTGTCAAAATAATTGTCCTGGTCAATTATTTGTAAAAGTTGCGCCTAATTTAACAAATGAATATGATCCTGATGCATCTGCTAATATGTCAAGAATTGTAACTTACTCAGATTTTTGGTGGAAAGGTAAATTAGTATTTAAAGCTAAACTAAGAGCATCTCATACTTGGAATCCAATTCAACAAATGAGTATTAATGTAGATAACCAATTTAACTATGTACCAAATAATATTGGAGCTATGAAAATTGTATATGAAAAATCTCAACTAGCACCTAGAAAATTATATTAACATACTTACTATGTTTTTATGTTTATTACATATTAACTAGCACCTAGAAAATTATATTAATATACTTACTATGTTTTTATGTTTATTACATATTATTTTAAGATTAATTAAATTACAGCATAGAAATATTGTACTTGTATTTGATATAGGATTTAGAAGGTTTGTTATATGGTATACAATAACTGTAAGAAATAGAAGAACATTTAGATCATAGTTAGTAGTTTGTTTTATAAAATGTATTGTAGACTATTAATGTATGTTGTTATGGTGTGGGTGGTTGGTTGGTTTGCCCTTAGAAT

Appendix 3: Whole genome of VP2 fragment of FPV Pallas's cats isolate ATGAGTGATGGAGCAGTTCAACCAGACGGTGGTCAACCTGCTGTCAGAAATGAAAGAGCTACAGGATCTGGGAACGGGTCTGGAGGCGGGGGTGGTGGTGGTTCTGGGGGTGTGGGGATTTCTACGGGTACTTTCAATAATCAGACGGAATTTAAATTTTTGGAAAACGGGTGGGTGGAAATCACAGCAAACTCAAGCAGACTTGTACATTTAAATATGCCAGAAAGTGAAAATTATAAAAGAGTAGTTGTAAATAATATGGATAAAACTTCAGTTAAAGGAAACATGGCTTTAGATGATACTCATGTACAAATTGTAACACCTTGGTCATTGGTTGATGCAAATGCTTGGGGAGTTTGGTTTAATCCAGGAGATTGGCAACTAATTGTTAATACTATGAGTGAGTTGCATTTAGTTAGTTTTGAACAAGAAATTTTTAATGTTGTTTTAAAGACTGTTTCAGAATCTGCTACTCAGCCACCAACTAAAGTTTATAATAATGATTTAACTGCATCATTGATGGTTGCATTAGATAGTAATAATACTATGCCATTTACTCCAGCAGCTATGAGATCTGAGACATTGGGTTTTTATCCATGGAAACCAACCATACCAACTCCATGGAGATATTATTTTCAATGGGATAGAACATTAATACCATCCCATACTGGAACTAGTGGCACACCAACAAATGTATATCATGGTACAGATCCAGATGATGTTCAATTTTATACTATTGAAAATTCTGTACCAGTGCACTTACTAAGAACAGGTGATGAATTTGCTACAGGAACATTTTTTTTTGATTGTAAACCATGTAGACTAACACATACATGGCAAACAAATAGAGCATTGGGCTTACCACCATTTTTAAATTCTTTGCCTCAATCTGAAGGAGCTACTAACTTTGGTGATATAGGAGTTCAACAAGATAAAAGACGTGGTGTAACTCAAATGGGAAATACAGACTATATTACTGAAGCTACTATTATGAGACCAGCTGAGGTTGGTTATAGTGCACCATATTATTCTTTTGAAGCATCTACACAAGGGCCATTTAAAACACCTATTGCAGCAGGACGGGGGGGAGCGCAAACAGATGAAAATCAAGCAGCAGATGGTGATCCAAGATATGCATTTGGTAGACAACATGGTCAAAAAACTACTACAACAGGAGAAACACCTGAGAGATTTACATATATAGCACATCAAGATACAGGAAGATATCCAGAAGGAGATTGGATTCAAAATATTAACTTTAACCTTCCTGTAACAAATGATAATGTATTGCTACCAACAGATCCAATTGGAGGTAAAACAGGAATTAACTATACTAATATATTTAATACTTATGGTCCTTTAACTGCATTAAATAATGTACCACCAGTTTATCCAAATGGTCAAATTTGGGATAAAGAATTTGATACTGACTTAAAACCAAGACTTCATGTAAATGCACCATTTGTTTGTCAAAATAATTGTCCTGGTCAATTATTTGTAAAAGTTGCGCCTAATTTAACAAATGAATATGATCCTGATGCATCTGCTAATATGTCAAGAATTGTAACTTACTCAGATTTTTGGTGGAAAGGTAAATTAGTATTTAAAGCTAAACTAAGAGCATCTCATACTTGGAATCCAATTCAACAAATGAGTATTAATGTAGATAACCAATTTAACTATGTACCAAATAATATTGGAGCTATGAAAATTGTATATGAAAAATCTCAACTAGCACCTAGAAAATTATATTAA

Appendix 4: Amino acid sequence of VP2 fragment of FPV Pallas's cats isolate MSDGAVQPDGGQPAVRNERATGSGNGSGGGGGGGSGGVGISTGTFNNQTEFKFLENGWVEITANSSRLVHLNMPESENYKRVVVNNMDKTSVKGNMALDDTHVQIVTPWSLVDANAWGVWFNPGDWQLIVNTMSELHLVSFEQEIFNVVLKTVSESATQPPTKVYNNDLTASLMVALDSNNTMPFTPAAMRSETLGFYPWKPTIPTPWRYYFQWDRTLIPSHTGTSGTPTNVYHGTDPDDVQFYTIENSVPVHLLRTGDEFATGTFFFDCKPCRLQKTTTTGETPERFTYIAHQDTGRYPEGDWIQNINFNLPVTNDNVLLPTDPIGGKTGINYTNIFNTYGPLTALNNVPPVYPNGQIWDKEFDTDLKPRLHVNAPFVCQNNCPGQLFVKVAPNLTNEYDPDASANMSRIVTYSDFWWKGKLVFKAKLRASHTWNPIQQMSINVDNQFNYVPNNIGAMKIVYEKSQLAPRKLY
